# The Eagle Has Landed: an unusual case of Eagle’s syndrome

**DOI:** 10.1093/jscr/rjab530

**Published:** 2021-12-11

**Authors:** Reem AlAbdulwahed, Paul O’Flynn

**Affiliations:** Department of Head and Neck Surgery, University College London Hospital, 235 Euston Rd, Bloomsbury, London NW1 2BU, UK; Department of Head and Neck Surgery, University College London Hospital, 235 Euston Rd, Bloomsbury, London NW1 2BU, UK

## Abstract

Eagle’s syndrome refers to a group of characteristic symptoms affecting the oropharynx and neck that are caused by regional compression of structures due to a calcified stylohyoid ligament or an elongated styloid process. Of its two types, the former type is more common than the latter, but carotid artery dissection as a result of an elongated styloid is exceptionally rare. Following an extensive literature review, we present a case of carotid artery dissection causing multiple strokes secondary to direct compression from an elongated styloid process. Managed surgically through excision of the elongated styloid and post-operative rehabilitation, the patient recovered well and was discharged on anti-thrombotic medication, preventing further potentially detrimental attacks.

## INTRODUCTION

Eagle’s syndrome refers to a group of characteristic symptoms affecting the oropharynx and neck caused by regional compression of structures due to a calcified stylohyoid ligament or an elongated styloid process. First discovered in 1937 by American otolaryngologist Watt W. Eagle, the eponymous syndrome’s symptoms fall under two categories: the ‘classic’ presentation, involving pharyngeal pain referred to the ear, associated with dysphagia and odynophagia. This generally occurs post-tonsillectomy due to development of scar tissue around the styloid. The second category, ‘stylocarotid artery syndrome’, is where an elongated styloid or calcified stylohyoid ligament compresses the internal or external carotid artery, causing pain along its course as a result of sympathetic chain stimulation [1]. It may lead to dissection of the vessel and ultimately, a transient ischaemic attack or stroke. The styloid is termed elongated if it exceeds 3 cm. This occurs in 4% of the population but is only termed Eagle’s syndrome if symptomatic, which is estimated at 4%, resulting in an actual incidence of 0.16%. The syndrome has a female predominance, occurring 3:1 between the ages of 30 and 50. It is known that the classic type is commoner than the stylocarotid type, but carotid artery dissection resulting from an elongated styloid is exceptionally rare [2, 3]. Following a literature review, we present a case of carotid dissection causing multiple strokes secondary to compression from an elongated styloid.

## CASE REPORT

A healthy 35-year-old man presented to his local A&E with a short history of left arm weakness and facial droop. He also complained of a right-sided headache and neck stiffness. On examination, his vitals were normal and glasgow coma scale (GCS) was 15/15. Neurological examination revealed mild left facial droop and a power of 4/5 in his left upper limb with sensory loss. Computed tomography brain did not show any infarction or bleeding. A lumbar puncture ruled out meningitis and subarachnoid haemorrhage. He later collapsed with left hemiplegia and was urgently transferred to a tertiary centre where magnetic resonance imaging confirmed multiple embolic infarcts in the right middle cerebral artery (MCA) territory. He was started on dual antiplatelet therapy as standard stroke management. Despite this, he developed two further episodes of hemiplegia with expressive aphasia. Repeat imaging confirmed new infarctions in the right MCA territory, a new infarction in the right anterior cerebral artery territory and a right M1 thrombus. In light of this, he underwent a thrombectomy that did not resolve his symptoms. Numerous blood tests such as anti-ENA, ANCA, HIV and antiphospholipid antibodies were negative. Echocardiography ruled out mural thrombi and a 7-day Holter monitor ruled out arrhythmias. Additional imaging revealed an elongated right styloid in close proximity to a 15-mm internal carotid artery (ICA) dissection, leading to a diagnosis of stylocarotid Eagle’s syndrome.

Treatment modalities vary according to the type of Eagle’s syndrome encountered but are conservative or surgical [3]. Conservative treatment for the classical type entails the use of non-steroidal anti-inflammatory medication, corticosteroids, anticonvulsants and antidepressants, similar to the treatment of trigeminal neuralgia and neuropathic pain [5], in addition to patient education on avoiding over-flexion and over-extension of the neck [6]. The latter is generally managed surgically, either intraorally or extraorally to resect the elongated styloid, both with high success rates of 93.4% [3, 5]. Considering the higher morbidity and complication risk with the intraoral approach such as deep neck space infection, neurovascular injury, airway oedema and the need for an ipsilateral tonsillectomy, most surgeons favour a pharyngeal approach through a transcervical incision [3–5]. The extraoral approach provides better visualisation of the operative field albeit comes with a price of longer operating time and a visible surgical scar. The risk of iatrogenic neurovascular injury cannot be eliminated either [5].

## DISCUSSION

**Figure 1:**
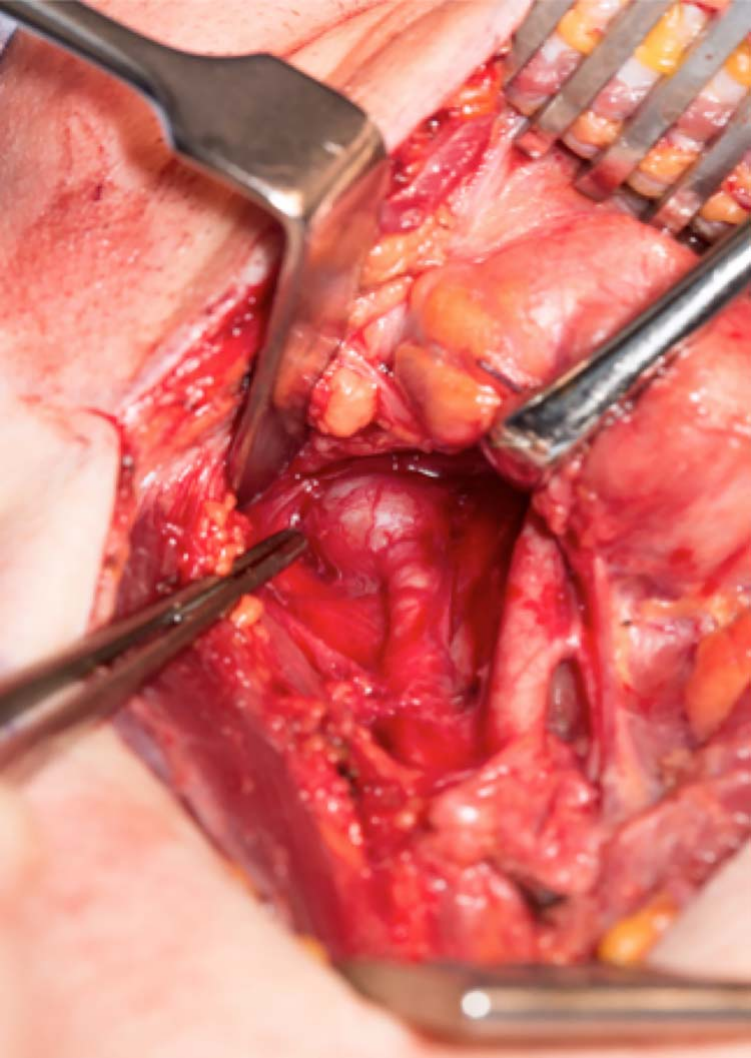
Internal carotid pseudoaneurysm as seen through a transcervical approach.

**Figure 2:**
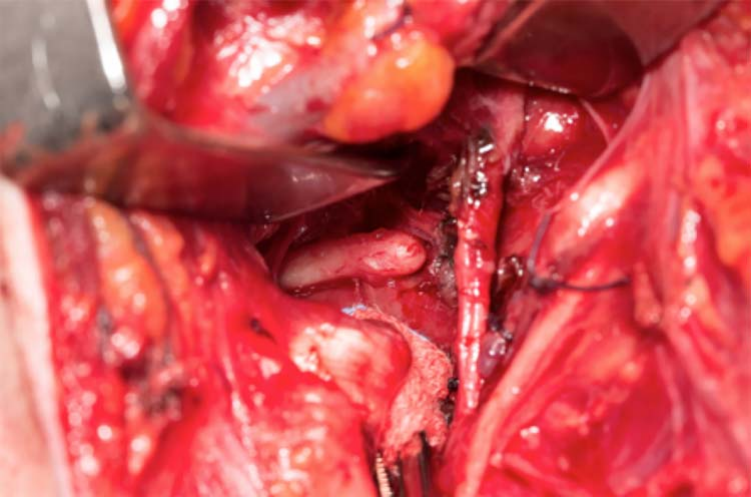
Elongated styloid seen through a transcervical approach.

**Figure 3:**
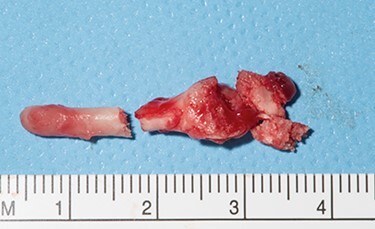
Excised styloid process measuring ~4 cm.

Sveinsson *et al*. (2013) [7] reported his literature review showed only a handful of cases of carotid dissection in context of an elongated styloid and even less so where a stroke resulted. Our patient suffered from three ischaemic strokes over a 12-day period. He underwent an unsuccessful thrombectomy prior to discovering the elongated styloid and was transferred to us for further management. Following a multi-disciplinary team meeting, he underwent a styloidectomy through a transcervical approach. After identification and retraction of the internal jugular vein, external carotid artery and hypoglossal nerve, an internal carotid artery pseudo-aneurysm was revealed (Fig. 1) with close relation to the tip of the styloid. The carotid artery was not stented during the procedure, to be managed conservatively and the styloid was trimmed measuring ~4 cm in length (Figs 2 and 3). Post-operatively, he developed mild pharyngeal oedema that resolved with a short course of intravenous steroids. He recovered well with the help of speech and physiotherapy, regaining full power in both his upper and lower limbs. His nutrition was gradually built up until he was tolerating a normal diet orally. He was discharged on dual anti-platelets 7 days later and continued rehabilitation in the community.

Shindo *et al*. (2019) described a case of carotid artery dissection caused by stylocarotid Eagle’s syndrome that was managed conservatively with no recurrent strokes at 18-month follow-up. Their paper argued that there may be a ‘surgical management’ publication bias when it comes to this type of Eagle’s syndrome, that conservative management must be considered as there is not enough data proving the direct causation of ICA dissections by the elongated styloid [6]. In our case, there were no positive factors identified to cause the dissection despite numerous investigations. That, with no medical history, led us to believe that the dissection must have been secondary to compression from the elongated styloid. Additionally, the length of our patient’s styloid exceeded that mentioned in their case. This could be an important element in the decision process to determine the best treatment plan. Conservative management may be appropriate should the case permit given the risks associated with surgery, but there is also a hefty morbidity associated with every new infarct should conservative management fail. This is a condition that has proved that a ‘one size fits all’ approach must be avoided. Whilst a conservative approach was successful for their patient, it was not an option for ours.

## CONFLICT OF INTEREST STATEMENT

None declared.

## FUNDING

None.
